# Higher Levels of Serum Zonulin May Rather Be Associated with Increased Risk of Obesity and Hyperlipidemia, Than with Gastrointestinal Symptoms or Disease Manifestations

**DOI:** 10.3390/ijms18030582

**Published:** 2017-03-08

**Authors:** Bodil Ohlsson, Marju Orho-Melander, Peter M. Nilsson

**Affiliations:** 1 Department of Internal Medicine, Skane University Hospital, Lund University, 205 02 Malmö, Sweden; peter.nilsson@med.lu.se; 2 Genetic Epidemiology, Diabetes and Cardiovascular Disease, Lund University, 205 02 Malmö, Sweden; marju.orho-melander@med.lu.se

**Keywords:** blood pressure, gastrointestinal disorders, gastrointestinal symptoms, hyperlipidemia, obesity, overweight, zonulin

## Abstract

Zonulin is considered a biomarker of increased intestinal permeability, and elevated levels have been found in celiac disease. The primary aim of this study was to examine the association between serum zonulin levels and gastrointestinal (GI) symptoms, and secondarily, between zonulin levels and anthropometric and metabolic factors. The offspring (*n* = 363) of the participants of the Malmö Diet and Cancer cardiovascular cohort (MDC-CV) were invited to an anthropometric and clinical examination, where fasting plasma glucose levels were measured. Questionnaires about lifestyle factors and medical history were completed along with the Visual Analog Scale for Irritable Bowel Syndrome (VAS-IBS). Zonulin levels were measured in serum by ELISA. Neither GI symptoms nor GI diseases had any influence on zonulin levels. Higher zonulin levels were associated with higher waist circumference (*p* = 0.003), diastolic blood pressure (*p* = 0.003), and glucose levels (*p* = 0.036). Higher zonulin levels were associated with increased risk of overweight (*p* < 0.001), obesity (*p* = 0.047), and hyperlipidemia (*p* = 0.048). We cannot detect altered zonulin levels among individuals reporting GI symptoms or GI diseases, but higher zonulin levels are associated with higher waist circumference, diastolic blood pressure, fasting glucose, and increased risk of metabolic diseases.

## 1. Introduction

The spaces between epithelial cells are controlled by at least four intercellular junctions that regulate the paracellular intestinal permeability. Tight junctions are the best well-described junctions, and these junctions are regulated by over 50 proteins [1]. Zonulin is the only measurable blood protein that reflects the intestinal permeability, and increased zonulin levels are considered to be a marker of impaired intestinal barrier [2,3]. Zonulin is the eukaryotic counterpart of the *Vibrio cholerae* zonula occludens toxin [4]. Human zonulin (47-kDa protein) is identical to prehaptoglobin-2, and binds to the epidermal growth factor receptor (EGFR) and protease-activated receptor 2 (PAR2) in the intestinal epithelium. This complex initiates phosphorylation of zonula occludens proteins, and leads to the small intestine’s tight junction disassembling [5]. Zonulin is secreted mainly from the liver, but also from enterocytes, adipose tissue, brain, heart, immune cells, lungs, kidney, and skin [6,7]. The secretion of zonulin is triggered by gluten and bacteria [2], and as zonulin increases the intestinal permeability, the ability for the washout of bacterial colonization in the gut is improved [8].

Gastrointestinal (GI) symptoms and diseases, especially irritable bowel syndrome (IBS), is very common in the population [9]. Today, we have no objective measurable marker to assess the degree of symptoms or complaints. Calprotectin in feces is used world-wide to discover mucosal inflammation, but is of no use to estimate symptom severity, or to identify functional bowel disorders [10]. Since half of the patients with diarrhea-predominated IBS have been shown to exhibit an increased intestinal permeability [11], the hypothesis is that serum zonulin could be of use to grade GI symptoms and diseases.

High serum levels of zonulin have been found in several autoimmune diseases, e.g., celiac disease and type 1 diabetes, which could explain the changed intestinal permeability observed in diabetes prior to detected intestinal complications [2,12]. Circulating zonulin has been shown to be elevated also in non-autoimmune diseases, e.g., in type 2 diabetes and obesity. Its concentration has been shown to correlate with glucose levels, dyslipidemia, inflammation, and insulin resistance [13,14]. The dietary influence on the zonulin secretion has shown contradictory results, and no definite conclusions have been able to be drawn [13,15,16,17].

Zonulin has previously only been considered an inactive precursor of haptoglobin, a protein secreted mainly by hepatocytes to act as a hemoglobin scavenger [18]. Haptoglobin binds to hemoglobin to form stable complexes, preventing oxidative tissue damage. In addition, haptoglobin has anti-oxidant and anti-inflammatory effects [7,18]. Thus, the presence of zonulin may not only reflect intestinal permeability [2], but may also reflect a reaction secondary to inflammation [19].

Since many factors have been supposed to be involved in the regulation of zonulin levels, we wanted to examine associations of zonulin levels in serum. The primary aim of this observational study was to examine the levels of zonulin in a population-based cohort and to analyze cross-sectional associations with GI factors. The secondary aims were to study the associations between zonulin and anthropometric and metabolic factors.

## 2. Results

### 2.1. Subject Characteristics

In the study, 363 subjects were included and their blood samples were collected, 177 (49%) males and 186 (51%) females. Of these, 238 (66%), had completed the study questionnaire and 235 (65%) the VAS-IBS as well. Higher age in those who completed the questionnaire (44 (30–54) years vs. 37 (26–52) years; *p* = 0.026) was the only difference observed in the measured parameters between those who completed or did not complete the questionnaires. Most subjects were middle-aged with normal body mass index (BMI), and with blood pressure and fasting glucose levels within reference ranges (Table 1). One-third of the subjects were living alone, and less than half of them had a university degree. The vast majority were working. A minority were smokers or had a high alcohol consumption. The majority had an occupation which required light physical activity, and had moderate to regular physical activity during their leisure time (Table 2).

Of the 238 participants who completed the VAS-IBS, 44 (18%) individuals had had GI symptoms during the last 2 weeks. Bloating and flatulence was the most pronounced symptom, followed by abdominal pain (Table 3). Functional dyspepsia was the most common GI disease (*n* = 42), followed by IBS (*n* = 40), and reflux (*n* = 33) (Table 4).

### 2.2. Zonulin Values

There was no difference in serum levels of zonulin between gender (*p* = 0.137). Weight, BMI, waist and hip circumference, blood pressure, and fasting plasma glucose levels correlated with zonulin levels (Table 1), whereas socio-economic factors and life style habits did not influence zonulin levels (Table 2). GI symptoms or psychological well-being did not correlate with serum values of zonulin (Table 3).

Subjects with any history of organic GI diseases, i.e., celiac disease, Crohn´s disease, lactose intolerance, reflux, ulcer, or ulcerative colitis (*n* = 54), had the same zonulin levels as those without (53.6 (47.9–63.4) ng/mL vs. 53.6 (44.0–62.7) ng/mL; *p* = 0.566), as was also true for those with any functional GI symptoms, i.e., functional dyspepsia or IBS (*n* = 61), compared with those without (52.7 (46.2–59.7) ng/mL vs. 53.7 (44.2–63.7) ng/mL; *p* = 0.530). Also, when only participants with present GI diseases, namely celiac disease, Crohn´s disease, and ulcerative colitis were calculated together (*n* = 7), there was no difference compared with healthy participants (57.6 (47.7–65.6) ng/mL vs. 53.6 (44.2–62.8 ng/mL; *p* = 0.476). The presence of GI symptoms during the last 2 weeks (*n* = 44) did not affect zonulin levels (52.3 (41.6–59.0) ng/mL vs. 54.5 (45.2–63.6) ng/mL; *p* = 0.297). Presence of diarrhea (*n* = 35) during the last 2 weeks did not affect zonulin levels (53.6 (47.2–59.1) ng/mL vs. 53.8 (45.0–63.5) ng/mL; *p* = 0.636).

When examining the associations of zonulin levels with continuous values such as age, gender, height, weight, BMI, waist and hip circumference, blood pressure, and fasting plasma glucose levels, all but age, gender, and height were associated with zonulin levels (Table 5). After adjusted calculations, higher waist circumference, diastolic blood pressure, and fasting glucose levels were independent risk markers of elevated zonulin levels (Table 5).

The calculation of differences between healthy individuals and subjects suffering from different risk conditions showed that subjects with overweight, obesity, hyperlipidemia and hypertension had higher zonulin levels compared with healthy subjects (Table 4).

When divided into participants suffering from overweight, obesity, diabetes, hyperlipidemia and/or hypertension (*n* = 143) or healthy (*n* = 220), the median values of zonulin were 56.8 (45.8–68.6) ng/mL vs. 53.0 (44.2–61.9) ng/mL (*p* = 0.008).

In a logistic regression model, higher zonulin levels were associated with increased risk to suffer from overweight (OR = 4.10 for highest compared with lowest quartile; 95% CI = 1.87–8.97), obesity (OR = 4.90 for highest compared with lowest quartile; 95% CI = 1.49–31.65), and hyperlipidemia (*p* for continuous values = 0.048), after adjustment for confounders (Table 6).

## 3. Discussion

The main findings of the present observational study in mostly middle-aged subjects were the associations between increased zonulin levels and increased waist circumference, diastolic blood pressure, and fasting plasma glucose levels. No associations between zonulin levels and GI symptoms or GI diseases could be detected in the present study. There were also associations between higher zonulin levels and increased risk of overweight, obesity, and hyperlipidemia.

The associations between zonulin levels and higher waist circumference, as well as elevated blood pressure and glucose levels, are in accordance with previous smaller studies which have shown that zonulin correlates with BMI, waist-to-hip ratio, plasma levels of glucose, cholesterol, triglycerides, tumor necrosis factor (TNF), systolic blood pressure, and insulin resistance [13,14,20]. Malyszko et al. [20] found a correlation between systolic blood pressure and zonulin, but patients with hypertension did not have higher levels compared with controls; these findings are in line with the present results when adjusted for confounders. The present weak association between zonulin levels and increased risk of hypertension and hyperlipidemia may depend on an under-powered study.

Zonulin is identical to prehaptoglobin-2 [5] and has been introduced as a serum biomarker reflecting intestinal permeability [2]. However, zonulin is secreted not only from enterocytes; it has been described in several extra-intestinal tissues, e.g., adipose tissue, brain, heart, immune cells, liver, lungs, kidney, and skin [6,7]. Thus, the levels of zonulin in serum do not only reflect intestinal secretion, but also secretion from other organs. Obese children with non-alcoholic fatty liver disease (NAFLD) had higher zonulin levels than obese children without NAFLD. The zonulin levels correlated with the degree of steatosis [16]. An increased release of zonulin from abdominal adipose tissue and liver may explain the elevated serum levels in overweight [6,7], with or without liver steatosis, and its correlation with waist circumference. For comparison, the liver is the main source of haptoglobin in serum [18]. In analogy with zonulin, haptoglobin levels have been shown to correlate with BMI and to be elevated in obesity [21]. Increased haptoglobin levels were found in subjects with elevated blood pressure, elevated glucose levels, or metabolic syndrome [22]. Thus, haptoglobin is considered a biomarker of these conditions [21,22]. Obesity and metabolic syndrome are considered to render a low-grade systemic inflammation [23]. Both zonulin and haptoglobin activate the complement system in vitro [24], and haptoglobin has been shown to be an independent determinant of CRP levels [21]. Altogether, the findings observed so far in the present and other studies of an association between increased levels of zonulin and elevated risk of overweight, obesity, and hyperlipidemia, suggest that elevated zonulin levels may rather be a biomarker of obesity, metabolic syndrome, and low-grade inflammation, than of increased intestinal permeability [3,13,14,16]. However, the degree of increased intestinal permeability during these conditions has to be determined, to try to identify the etiology behind and source of the elevated zonulin levels.

Zonulin has been reported to regulate tight junctions and intestinal permeability [5]. Nonetheless, more than 50 different proteins are participating in this regulation, and caution must be taken before considering serum zonulin as a sole biomarker of intestinal permeability [1]. To our knowledge, a direct comparison between zonulin and the lactulose:mannitol test has only been performed in one human study that examined patients with type 1 diabetes where zonulin measured as ng/mg total protein was correlated with urine concentration of the sugar probes [3]. Since patients with type 1 diabetes often display both low protein values in serum, hyperlipidemia, overweight, hypertension, and an impaired kidney function, several confounders may have influenced the results. In addition, obese subjects have an elevated glomerular filtration rate (GFR), and this may be reflected in a higher amount of excreted glucose as a consequence of glomerular hyperfiltration, rather than being a consequence of high intestinal absorption [25]. Accordingly, zonulin did not correlate with the lactulose:mannitol ratio in a rat model [26]. The assumption from epidemiologic and cross-sectional studies that zonulin levels reflect the degree of intestinal permeability [2,12,13,14] must be further examined in healthy subjects under well controlled conditions. We have previously demonstrated that there is no correlation between serum and feces levels of zonulin [17]. Thus, feces zonulin may be more associated with intestinal permeability, since secretion of zonulin from the intestinal barrier my leak into the lumen, whereas serum zonulin originates from several different tissues [6,7].

Apart from regulating the intestinal epithelium, zonulin also participates in the regulation of extra-intestinal epithelia and vascular endothelium, which also contain tight junctions [27]. Zonulin is of importance in regulation of permeability across all endothelial and epithelial surfaces, e.g., brain, intestinal epithelium, and lung tissue [2,24,28]. Hyperglycemia causes tissue damage and fosters the development of endothelial dysfunction [29,30]. This endothelial dysfunction strictly correlates with insulin resistance and inflammation [31]. The correlation between zonulin and blood pressure and glucose levels in this population-based cohort consisting of mainly healthy persons may indicate that blood pressure, and thereby the vascular tension and glucose and insulin concentrations may induce and/or regulate zonulin secretion from the blood vessels also during physiological conditions.

Although some correlations between zonulin and food components and excess energy intake have been described by some authors [13], this has not been found by others [15,16]. This depends on overweight, blood pressure, and lipid values that may interfere with effects induced by food components, and must be adjusted for when evaluating dietary factors. A dietary intervention in type 2 diabetes rendered higher zonulin levels in serum, albeit improved anthropometric and metabolic parameters [17]. The present study did not adjust for dietary factors, which is a limitation. The zonulin levels in general were higher in the present study compared with the data from the manufacturer when only 40 subjects were examined.

The degree of correlation between zonulin and inflammation and between zonulin and epithelial permeability still remains to be determined. Nonetheless, the conclusion from this and other studies in the field indicate elevated zonulin levels to be a biomarker of poor health. Zonulin could possibly be used clinically as a marker of the metabolic syndrome in the same way as described previously for the mature haptoglobin molecule [21,22]. Zonulin may be secreted secondary to impaired metabolism and exert anti-inflammatory effects, protecting the tissue from injury, in the same way as haptoglobin does [7]. Activation of the complement system and increased endothelial/epithelial permeability facilitates leakage of inflammatory cells into the damaged tissue [8,24]. Thus, zonulin secretion may reflect a protective mechanism and must not be a toxic product by itself. Further research has to determine the effect of zonulin on the endothelial and epithelial functions.

One limitation of the present study is the small study cohort, with few subjects suffering from GI symptoms or GI diseases. However, the prevalence of GI symptoms was in the magnitude as could be expected in a cohort representing the general population [9]. A larger cohort of subjects with GI diseases could possibly have revealed altered serum levels of zonulin in this cohort. Nevertheless, this small cohort could show statistically significant differences between patients with obesity and hyperlipidemia, although these conditions were present in fewer subjects, suggesting that serum zonulin is more important to reflect metabolic factors than GI factors. The hypothesis that serum zonulin could be used in the clinical practice to detect and follow patients with GI symptoms is not supported by the present findings.

## 4. Material and Methods

The subjects were treated according to the Helsinki declaration and the study was approved by the Regional Ethics Review Board at Lund University (Ref No 2012/594; 5 December 2012), Lund, Sweden. All subjects gave their written, informed consent before entering the study.

### 4.1. Study Subjects

All men and women living in Malmö, Sweden between 1991 and 1996, born between 1923 and 1950, were invited to participate in a population-based prospective cohort study. This cohort constitutes the Malmö Diet and Cancer Study (MDCS) [32]. Altogether, 28,098 participants completed all of the baseline examinations. From this cohort, 6103 individuals were randomly selected and comprises the Malmö Diet and Cancer cardiovascular cohort (MDC-CV). We plan to invite most of the offspring (children and grand-children) to subjects participating in the MDC-CV study cohort at baseline (*n* = 6103), after identification based on official register information. At the time of the present examination, 1500 subjects had attended (participation rate 45%).

### 4.2. Study Design

Subjects were invited to an anthropometric and clinical examination including measurement of weight (kg) and height (m) in light indoor clothing, waist and hip circumference (cm), blood pressure (mm·Hg) (Omron^®^ automatic reading, after 10 min supine rest and 5 min standing, mean of two readings), and pulse rate (beats and min) at the Clinical Research Unit (CRU), Skåne University Hospital, Malmö, Sweden. All participants had to complete a study questionnaire including questions on family history, socio-economy, lifestyle factors, medical history, and the Visual Analog Scale for Irritable Bowel Syndrome (VAS-IBS) with questions about GI symptoms. Fasting blood samples were collected for measurements of glucose in plasma and zonulin in serum.

### 4.3. Questionnaires

#### 4.3.1. Study Questionnaire

This questionnaire included questions on family history, medical history, lifestyle, educational achievement, intellectual and cultural activities, stress, self-perceived age, and social factors. This questionnaire is web-based and linked in structure and design to more or less similar questionnaires used by other large current population-based and on-going screening projects in Sweden (i.e., LifeGene, EpiHealth, BIG-3, SCAPIS).

#### 4.3.2. Visual Analog Scales for Irritable Bowel Syndrome

The Visual Analog Scale for Irritable Bowel Syndrome (VAS-IBS) was used to investigate GI complaints. It is a validated questionnaire for estimation of the most common GI complaints that patients with non-organic, functional bowel disease experienced during the previous 2 weeks [33]. This scale has also been validated for estimation of symptoms over time [34]. The seven items measured in the VAS-IBS address the symptoms of abdominal pain, diarrhea, constipation, bloating and flatulence, nausea and vomiting, psychological well-being, and intestinal symptoms´ influence on daily life. These items were measured on a scale from 0–100, where 0 represents severe problems and 100 represents a complete lack of problems.

### 4.4. Blood Sampling

All samples consisted of whole blood and were drained into ethylenediaminetetra-acetic acid (EDTA) glass tubes (BD Microtainer, Franklin Lakes, NJ, USA) or into serum separate tubes (SST) (BD Microtainer) in the morning after a 10-h fast. Measurements of glucose were performed from blood in EDTA tubes by HemoCue^©^ (HemoCue AB, Ängelholm, Sweden). Plasma glucose is equivalent to capillary measured glucose. The other samples were immediately cooled and centrifuged at 3000 rcf for 10 min. Serum was harvested and stored at −80 °C until analyzed for the concentrations of zonulin within 4 months.

### 4.5. Zonulin Measurement

The zonulin concentration was determined by using an ELISA kit (Immundiagnostik AG, Bensheim, Germany, batch No. K5601-150513). The assay used the competitive binding technique. Biotinylated zonulin tracer was added to the samples, standards, and controls as a competitor to the sample’s own zonulin. The intensity of the color was inver¬sely proportional to the zonulin concentration in the sample. Samples were read at 450 nm, and the 4-parameter algorithm was used to form the standard curve and to calculate data. All tests were carried out in duplicate. Zonulin concentration is presented in ng/mL. Based on the manufacturer´s studies of serum samples of apparently healthy persons (*n* = 40), a mean value of 34 ± 14 ng/mL was estimated. Inter-assay coefficient of variance (CV) were 13.3% and 13.6% for the lowest and highest control, respectively, and intra-assay CV were 3.4% and 6.0% for the lowest and highest control, respectively.

### 4.6. Data Categorization

Educational level was divided into primary school, upper secondary school, and university degree. Occupation was divided into working, retirement, student, sick leave, and unemployed. Marital status was divided into single and cohabitation. Subjects were classified to suffer from over-weight when BMI was ≥25.0 kg/m^2^, and from obesity when BMI was ≥30.0 kg/m^2^ [35]. The individual symptoms in the VAS-IBS scale was defined to be present when the score was below median values of reference values in healthy controls [36].

Smoking habits were categorized into four groups: never smokers (or less than 100 cigarettes in total), regular smokers, sporadic smokers, and former smokers. The frequency of alcohol drinking was categorized into never, ≤1 time per month, 2–3 times per month, 2–3 times per week, or ≥4 times per week. The amount of standard drinks on such a typical day was divided into nothing, 1–2 glasses, 3–4 glasses, 5–6 glasses, 7–9 glasses or ≥10 glasses. Occupational activity was set to very light, light, moderate heavy, and very heavy. Activity during leisure time was divided into mostly sitting, moderate activity, regular exercise, and regular training. Self-reported stress was divided into stress during the previous year and during the last 5 years.

### 4.7. Statistical Analyses

The data was analyzed using the software SPSS, IBM, North Castle, NY, USA, version 23.0 for Windows.

The continuous variables were not normally distributed and were therefore categorized and presented as medians (interquartile ranges (IQR)). Zonulin levels were also logarithmically transformed and categorized into above or beneath median value. Spearman´s correlation test was used for correlations between zonulin and continuous variables, and Kruskal-Wallis test or Mann-Whitney U-test were used for calculations of differences in continuous variables between groups. Fisher´s exact test was used to calculate differences in dichotomous variables between groups.

Factors intended to study (independent variables) for influence on zonulin levels, namely, age, gender, height, weight, BMI, waist and hip circumference, systolic and diastolic blood pressure, and fasting glucose levels, were initially examined using an unconditional logistic regression to calculate odds ratios (OR) with 95% confidence interval (CI). The reference was set to the lowest category of each variable. Analyses were then performed adjusted for variables with statistical significance according to the initial calculation, i.e., weight, BMI, waist and hip circumference, systolic and diastolic blood pressure, and fasting glucose levels.

In disorders characterized by elevated zonulin levels compared with healthy subjects, the influence of zonulin on disease was further examined with unconditional logistic regression to calculate OR with 95% CI, followed by adjustment for independent confounders. Over-weight and obesity were adjusted for diastolic blood pressure and fasting glucose levels; hypertension was adjusted for waist circumference and fasting glucose levels; hyperlipidemia was adjusted for waist circumference, diastolic blood pressure and fasting glucose levels; and diabetes was adjusted for waist circumference and diastolic blood pressure. In the statistical calculations, the definition of over-weight included all subjects with BMI ≥ 25 kg/m^2^ and the definition of obesity included all subjects with BMI ≥ 30 kg/m^2^. A *p*-value < 0.05 was considered statistically significant.

## 5. Conclusions

In this population-based study, we could not detect altered zonulin levels among individuals reporting GI symptoms or GI diseases. On the other hand, higher zonulin levels are associated with higher waist circumference, diastolic blood pressure, fasting glucose levels, increased risk of overweight, obesity, and hyperlipidemia. These data suggest that serum zonulin levels are more dependent on metabolic conditions than on GI diseases.

## Figures and Tables

**Table 1:** Subject characteristics and their correlations with zonulin levels.

Variable	Median (Interquartile)	Correlation Coefficient	*p*-Value
Age (year)	43 (28–53)	0.083	0.114
Height (cm)	173 (167–182)	0.025	0.640
Weight (kg)	78 (67–90)	0.193	<0.001
BMI (kg/m^2^)	22.3 (19.8–25.3)	0.213	<0.001
Waist circumference (cm)	89 (79–97)	0.271	<0.001
Hip circumference (cm)	104 (99–110)	0.173	0.001
Blood pressure (mm·Hg)			
Systolic	115 (106–127)	0.120	0.024
Diastolic	72 (65–77)	0.178	0.001
Plasma glucose (mmol/L)	5.3 (4.9–5.7)	0.138	0.009
Serum zonulin (ng/mL)	54.5 (45.2–64.4)		

Number of subjects = 363. Values are presented as median and interquartile range. Spearman’s correlation test. *p* < 0.05 was considered statistically significant.

**Table 2:** Study population characteristics.

Variable	Prevalence of Condition Yes/No	*p*-Value
Marital status (single/cohabitant)	77/161	0.904
Education		0.750
Primary school	12/226	
Upper secondary school	123/115	
University degree	102/136	
Occupation		
Working	192/40	0.499
Retirement	12/194	0.893
Student	23/188	0.566
Sick leave	8/200	0.144
Unemployed	10/197	0.901
Smoking		0.849
Never smokers	142/92	
Regular smokers	16/218	
Sporadic smokers	18/216	
Former smokers	58/176	
Alcohol (frequency of drinking)		0.740
Never	14/219	
Once monthly or less	44/189	
2–3 times a month	87/146	
2–3 times a week	82/151	
≥4 times a week	6/227	
Alcohol consumption (volume of drinking at each occasion)		0.951
1–2 glasses	121/97	
3–4 glasses	66/152	
5–6 glasses	20/198	
7–9 glasses	9/209	
≥10 glasses	2/216	
Occupational activity		0.467
Very light	106/118	
Light	36/188	
Moderate heavy	44/180	
Heavy	29/195	
Very heavy	9/115	
Leisure time activity		0.441
Mostly sitting	19/215	
Moderate activity	95/139	
Regular exercise	60/174	
Regular training	60/174	
Stress during the last year	102/132	0.346
Stress during the last 5 years	90/143	0.936

Number of subjects = 238. Kruskal-Wallis or Mann-Whitney U test were used to calculate differences in zonulin levels between groups. *p* < 0.05 was considered statistically significant.

**Table 3:** Correlations between zonulin levels and gastrointestinal symptoms during the last 2 weeks.

Symptom	Median (IQR) (mm)	Correlation Coefficient	*p*-Value	Symptom Prevalence (n)
Abdominal pain	64 (39–89)	0.035	0.828	37/195
Diarrhea	80 (40–93)	−0.064	0.691	35/196
Constipation	92 (45–100)	−0.103	0.521	20/211
Bloating and flatulence	56 (37–87)	−0.082	0.607	30/202
Nausea and vomiting	96 (72–100)	0.125	0.444	22/208
Psychological well-being	80 (40–94)	0.153	0.334	180/41
Intestinal symptoms’ influence on daily life	68 (41–91)	−0.103	0.516	34/198

Total number of subjects with gastrointestinal symptoms = 44, out of 235 completed questionnaires. Values of Visual Analog Scale for Irritable Bowel Syndrome (VAS-IBS) are presented as median and interquartile range (IQR) in mm. The items were measured on a scale from 0–100, where 0 represents severe problems and 100 represents a complete lack of problems. The individual symptom was considered to be present when the item scale score was below the median value in healthy controls [ref No 36]. Spearman´s correlation test. *p* < 0.05 was considered statistically significant.

**Table 4:** Zonulin levels in relation to drug intake and various disease conditions.

Variable	Prevalence of Condition (Yes/No)	Median and Interquartile Values of Zonulin	*p*-Value
Over-weight (BMI ≥ 25 kg/m^2^)	99/264	60.1 (50.8–72.3)/52.1 (42.0–61.9)	<0.001
Obesity (BMI ≥ 30 kg/m^2^)	25/338	68.4 (55.6–85.2)/53.6 (44.2–63.5)	<0.001
Drugs with receipts	86/149		0.406
Drugs without receipts	41/191		0.359
Antibiotic use last 6 months	30/203		0.626
Morbidity			
Celiac disease	4/231		0.479
Lactose intolerance	12/221		0.502
Reflux	33/203		0.462
Ulcer	16/221		0.519
Functional dyspepsia	42/195		0.955
Irritable bowel syndrome	40/197		0.126
Crohn’s disease	3/234		0.832
Ulcerative colitis	2/234		0.953
Functional gastrointestinal diseases	61/176		0.530
Organic gastrointestinal diseases	54/183		0.566
Gastrointestinal symptoms last 2 weeks	44/191		0.297
Asthma bronchialis	30/206		0.303
Atrial fibrillation	2/235		0.228
Diabetes	11/225		0.108
Heart failure	1/236		0.540
Hyperlipidemia	32/202	58.1 (50.7–74.4)/53.2 (44.1–61.9)	0.004
Hypertension	49/189	56.4 (50.3–65.7)/52.1 (43.8–61.9)	0.021
Inflammatory joint disease	3/232		0.696
Malignancy	5/230		0.823
Myocardial infarction	2/236		0.873
Stroke	2/236		0.070

The number of subjects was 363 in the variables over-weight and obesity and 238 in other variables. Differences between healthy and diseased subjects were calculated by Mann-Whitney U test. *p* < 0.05 was considered statistically significant. Median and interquartile values are presented for conditions with statistically significant differences. Functional dyspepsia and irritable bowel syndrome constitute functional gastrointestinal disorders, and the other gastrointestinal diseases constitute the organic diseases.

**Table 5:** Subject characteristics in relation to low and high zonulin levels.

Variable	Crude OR	95% CI	*p*-Value	Adjusted OR	95% CI	*p*-Value
Age (year)						
<29	1.00					
29–43	1.16	0.650–2.08	0.613			
44–53	1.24	0.70–2.23	0.463			
>53	1.30	0.73–2.30	0.379			
Gender (177 male/186 female)	1.51	1.00–2.28	0.052			
Height (cm)						
<168	1.00					
168-173	1.06	0.59–1.88	0.857			
174-182	1.52	0.84–2.76	0.171			
>182	1.12	0.63–2.01	0.699			
Weight (kg)						
<68	1.00			1.00		
68–78	1.11	0.61–2.00	0.733	1.32	0.46–3.76	0.605
79–90	1.23	0.68–2.23	0.491	0.88	0.22–3.43	0.851
>90	2.67	1.46–4.87	0.001	0.58	0.11–3.12	0.521
BMI (kg/m^2^)						
<19.9	1.00			1.00		
19.9–22.3	0.86	0.47–1.55	0.607	0.51	0.17–1.54	0.231
22.4–25.3	1.25	0.70–2.24	0.451	0.75	0.16–3.43	0.713
>25.3	2.90	1.57–5.33	0.001	1.24	0.18–8.60	0.828
Waist circumference (cm)						
<80	1.00			1.00		
80–89	0.97	0.53–1.76	0.920	1.28	0.52–3.11	0.592
90–97	1.31	0.73–2.37	0.369	1.86	0.66–5.24	0.239
>97	4.76	2.54–8.90	<0.001	7.03	1.97–25.11	0.003
Hip circumference (cm)						
<100	1.00			1.00		
100–104	1.49	0.81–2.72	0.199	1.23	0.58–2.60	0.593
105–110	1.43	0.81–2.54	0.219	0.99	0.42–2.35	0.985
>110	2.57	1.43–4.62	0.002	0.81	0.26–2.48	0.708
Systolic blood pressure (mm Hg)						
<107	1.00			1.00		
107–115	1.84	1.00–3.37	0.049	1.31	0.64–2.66	0.457
116–127	1.72	0.96–3.10	0.070	1.12	0.48–2.61	0.801
>127	1.59	0.87–2.92	0.130	0.91	0.34–2.45	0.848
Diastolic blood pressure (mm Hg)						
<66	1.00			1.00		
66–72	2.92	1.57–5.42	0.001	2.82	1.43–5.58	0.003
73–77	2.56	1.39–4.69	0.002	1.58	0.72–3.48	0.258
>77	2.01	1.09–3.72	0.026	1.21	0.48–3.07	0.690
Plasma Glucose levels (mmol/L)						
<5.0	1.00			1.00		
5.0–5.3	1.05	0.57–1.95	0.872	1.05	0.53–2.09	0.890
5.4–5.7	1.26	0.73–2.18	0.404	0.97	0.53–1.80	0.926
>5.7	2.92	1.58–5.39	0.001	2.09	1.05–4.18	0.036

Number of subjects = 363. Logistic regression to examine the variables influence on zonulin levels, which were divided into low and high values according to the median value. Values are presented as median and interquartile values. OR = odds ratio, CI = confidence interval.

**Table 6:** Association of zonulin levels and risk conditions and disease categories.

Disease	Case/Control	Crude OR	95% CI	*p*-Values	Adj OR	95% CI	*p*-Values
Overweight (*n* = 99) (BMI ≥ 25 kg/m^2^)							
6.4–45.2	12/78	1.00			1.00		
45.3–54.4	20/71	1.83	0.84–4.01	0.131	1.63	0.72–3.73	0.245
54.5–64.3	28/63	2.89	1.36–6.14	0.006	2.36	1.07–5.21	0.033
64.4–133.4	39/52	4.88	2.34–10.18	<0.001	4.10	1.87–8.97	<0.001
P for trend				<0.001			<0.001
P for logarithmic value				<0.001			0.003
Obesity (*n* = 25) (BMI ≥ 30 kg/m^2^)							
6.4–45.2	2/88	1.00			1.00		
45.3–54.4	1/90	0.49	0.04–5.49	0.562	0.32	0.04–5.41	0.365
54.5–64.3	8/83	4.24	0.88–20.55	0.073	3.06	0.74–17.72	0.178
64.4–133.4	14/77	8.00	1.76–36.32	0.007	4.90	1.49–31.65	0.047
P for trend				<0.001			0.003
P for logarithmic value				<0.001			0.003
Hyperlipidemia (*n* = 32)							
6.4–45.2	3/53	1.00			1.00		
45.3–54.4	7/58	2.13	0.52–8.67	0.290	1.59	0.36–7.00	0.537
54.5–64.3	10/50	3.53	0.92–13.59	0.066	2.36	0.56–9.99	0.244
64.4–133.4	12/41	5.17	1.37–19.54	0.015	2.75	0.63–11.99	0.179
P for trend				0.005			0.117
P for logarithmic value				0.002			0.048
Hypertension (*n* = 49)							
6.4–45.2	6/52	1.00			1.00		
45.3–54.4	13/53	2.13	0.75–6.02	0.155	2.16	0.75–6.21	0.152
54.5–64.3	16/45	3.08	1.11–8.54	0.031	2.50	0.88–7.16	0.086
64.4–133.4	14/39	3.11	1.10–8.82	0.033	1.98	0.66–5.98	0.227
P for trend				0.023			0.229
P for logarithmic value				0.103			0.472
Diabetes (*n* = 11)							
6.4–45.2	2/55	1.00			1.00		
45.3–54.4	1/65	0.42	0.04–4.79	0.487	0.37	0.03–4.59	0.441
54.5–64.3	4/56	1.96	0.35–11.16	0.446	1.11	0.17–7.12	0.911
64.4–133.4	4/49	2.25	0.39–12.80	0.362	0.92	0.14–5.86	0.926
P for trend				0.118			0.573
P for logarithmic value				0.120			0.509

The number of subjects was 363 for the variables overweight and obesity and 238 for other variables. Zonulin values (ng/mL) were divided into quartiles or logarithmic for continuous values. The logistic regression adjusted for diastolic blood pressure (mm·Hg), fasting glucose levels, and waist circumference (cm). In the statistical calculations, the definition of overweight included all subjects with BMI ≥ 25 kg/m^2^ and the definition of obesity included all subjects with BMI ≥ 30 kg/m^2^. Overweight and obesity were only adjusted for diastolic blood pressure (mm·Hg) and fasting glucose levels; hypertension was only adjusted for waist circumference and fasting glucose levels; and diabetes was only adjusted for waist circumference and diastolic blood pressure. OR = odds ratio, CI = confidence interval.
